# Height After Side: Goalkeepers Detect the Vertical Direction of Association-Football Penalty Kicks From the Ball Trajectory

**DOI:** 10.3389/fpsyg.2020.00311

**Published:** 2020-02-27

**Authors:** Alfredo Higueras-Herbada, José E. Lopes, David Travieso, Jorge Ibáñez-Gijón, Duarte Araújo, David M. Jacobs

**Affiliations:** ^1^Facultad de Psicología, Universidad Autónoma de Madrid, Madrid, Spain; ^2^Municipality Sports Division, Ponte de Sor, Portugal; ^3^CIPER, Faculdade de Motricidade Humana, Universidade de Lisboa, Cruz Quebrada, Portugal

**Keywords:** ball height, goalkeeper movement, movement initiation, kinematic variables, penalty outcome

## Abstract

The present research analyzes the relation between the height of penalty kicks in association football and (a) the probability that goalkeepers stop the ball, (b) the kinematics of the kicker, and (c) the movements of the goalkeeper. We re-analyzed movement registration data that were collected in an experiment (with professional and semi-professional players) that focused on the horizontal direction of the penalties (Lopes et al., 2014). We also digitized and analyzed regular videos of the goalkeepers that were recorded by Lopes et al. (2014) but not analyzed. The present research complements the current understanding of the penalty kick with three main observations. First, goalkeepers save penalties at middle heights more often than low and high penalties. Second, the height of penalties is predicted less clearly than their horizontal direction from the kinematics of penalty takers. Third, goalkeepers tend to initiate the horizontal component of the saving action before the penalty taker contacts the ball, but they initiate the vertical component of the action about 245 ms after the contact. Taken together, these results support the view that goalkeepers make the left-right decision at least partly focusing on the kinematics of the kicker, and that they dynamically decide the vertical aspects of the movement later, focusing on the ball trajectory.

## Introduction

In association football, the goalkeepers’ probability to avoid a goal in a penalty situation is highly dependent on the direction of the ball (Bar-Eli and Azar, 2009) – among several other issues, such as the goalkeepers’ displacement capacity and body height (Dicks et al., 2010) and the timing of the saving action (van der Kamp et al., 2018). How do goalkeepers perceive the direction of penalty kicks and control their saving actions accordingly? The answer to this question depends on the aspect of the direction that one has in mind. Goalkeepers do not perceive and control the horizontal and vertical aspects of the penalty situation in the same way. A full understanding of the saving action requires a consideration of both dimensions.

Most research has focused on the horizontal direction of the penalty kicks (Franks and Harvey, 1997; Dicks et al., 2010a; Lees and Owens, 2011; Diaz et al., 2012; Lopes et al., 2014). For the horizontal direction, time constraints mandate that goalkeepers should not wait until after the moment of ball contact. Indeed, goalkeepers typically initiate their action before ball contact (McMorris and Hauxwell, 1997). To enhance the probability of choosing the correct side, goalkeepers rely on anticipatory information from the biomechanics of the penalty taker, probably complemented with situational information about the preferred shooting side of the opponent (Navia et al., 2013). Biomechanical variables that covary with the horizontal direction of penalty kicks include the non-kicking foot angle, knee angle of the kicking leg, speed of the kicking foot, kicking foot angle, hip angle, and movement direction of the kicking foot (Lees and Owens, 2011; Diaz et al., 2012; Lopes et al., 2014).

In addition to the horizontal direction, the height of penalty kicks is relevant in the sense that it exerts a strong influence on their outcome (Bar-Eli and Azar, 2009). The percentage of correct anticipation from the biomechanics of the penalty taker is substantially lower for the vertical than for the horizontal direction (McMorris et al., 1993; Savelsbergh et al., 2005; Murgia et al., 2014; Causer et al., 2017). In addition, the improvement with learning is less for the vertical direction (cf. McMorris and Hauxwell, 1997; Poulter et al., 2005). Particularly interesting are the results of Williams and Burwitz (1993). These authors reported that the percentage of errors in judging the location of penalty kicks that was attributable to the height aspect of the judgments was high (between 67 and 71%) when videos of penalty takers were occluded at 120, 40, or 0 ms before the moment of ball contact. This percentage dropped remarkably (to 41%) when the first 40 ms of the ball trajectory was shown. On the basis of these results, Williams and Burwitz (1993) recommended goalkeepers to use the initial part of the ball trajectory to adjust the height of their saving action.

An obvious aspect that has to be mentioned with regard to the relative difficulty to perceive the height of penalty kicks is that standard football goals have a width of 7.32 m and a height of 2.44 m. The limited variability in the height of penalties, and the associated difficulty in detecting height, have led several authors to claim that “postural cues relating to the height of the penalty kick are more subtle and harder to pick up than those responsible for conveying the correct side” (Savelsbergh et al., 2002, p. 284–285) and that “critical cues for determining ball height may not be available until late in the moment, or as suggested in previous research, until the first portion of ball flight is visible” (Causer et al., 2017, p. 8). We are not aware of studies on the biomechanics of penalty takers that confirm that the height of penalty kicks is more difficult to predict than the horizontal direction.

At coaching level it is widely accepted that kickers should lean forward or backward depending on whether they want to direct the ball to a lower or higher location. Williams and Burwitz (1993) showed that soccer players who are asked to anticipate the direction of penalty kicks share the belief that the trunk angle is crucial. Participants in their above-mentioned experiment judged this variable to be the most important predictor of the height of penalties. Prassas et al. (1990) analyzed biomechanical differences for low and high kicks (not penalty kicks, hence having different task constraints; Araújo et al., 2006). In this study, the mean backward lean was 17.5° for high kicks and 13.3° for low kicks. This difference was significant, providing at least partial support for the coaching recommendation concerning forward or backward lean. However, Prassas et al. (1990) reported significant differences for a substantial number of other variables, related to the kicking foot and leg, the non-kicking foot and leg, and the trunk and hip segments. Their overall conclusion was that the main determinant of performing a low or high kick is the height at which the ball is contacted with respect to its horizontal midline.

In sum, our knowledge about the behavior of penalty takers and goalkeepers is more substantial for the horizontal than for the vertical direction. In part this is so because research that relates the biomechanics of kickers to height has only been performed with kicks other than the penalty kick. To date, the movements of the goalkeeper have not been analyzed with regard to height. The present study addressed the height dimension in the specific case of the penalty kick. We used data from an experiment reported in Lopes et al. (2014). In the analyzed experiment, twelve players took 60 penalties each, using a standard size goal and a standard distance. The movements of the penalty takers were registered with movement-registration equipment. The original analyses focused on the biomechanics of the kicker in relation to the horizontal direction. In the present research, we supplemented those analyses with analyses on height and on the time at which goalkeepers initiate the horizontal and vertical aspects of their saving action.

Our analyses can be divided into three parts. First, we determined the efficacy of penalties shot at different heights, expecting to replicate that the height of a penalty is related to its outcome. Second, we used the movement-registration data from the kickers to determine the predictive value of different kinematic variables with respect to height. We expected height to be more difficult to predict from the body kinematics than the horizontal direction, and we expected the height of the kicking foot and the trunk angle at ball contact to be among the better predictors. Third, we analyzed the regular video recordings of the goalkeepers to determine when their hand positions diverge for penalties shot to the left and right and for penalties shot low and high. We expected the positions to diverge before ball contact for the horizontal direction and substantially later for the vertical direction, reflecting that goalkeepers’ decisions occur substantially later for the vertical than for the horizontal direction.

## Materials and Methods

### Participants

The participants in the experiment that we further analyzed (Lopes et al., 2014) were twelve male professional and semi-professional field players (*M*_age_ = 21.2 years; *SD* = 4.6 years) and five young but experienced non-professional goalkeepers from the same football club (*M*_age_ = 17.4 years; *SD* = 0.9 years). All participants played in the Portuguese National Second Division or in the Portuguese National Junior Second Division. Informed consent was obtained from the players and from their club, after the ethical approval of the study by a local university committee. For the minors, the informed consent was obtained from their parents.

### Materials

An indoor setting was used. Pieces of green and red tissue that spanned the full height of the goal were placed at the left and right sides of the goal, respectively. The experiment was recorded with a standard video camera (25 Hz; DCR-HC23, Sony Corporation, Tokyo, Japan) and a four-camera infrared system (150 Hz; Qualisys AB, Gothenburg, Sweden). The infrared system recorded 12 markers that were attached to the head, shoulders, elbows, wrists, hips, and knees; and four markers attached to the backside and outer side of each shoe.

### Design and Procedure

Trials differed with regard to the side of the penalty (left/green vs. right/red) and with regard to the deception condition (with vs. without deception). This led to the following instructions: “shoot to green without simulating,” “shoot to red without simulating,” “shoot to green but simulate shooting to red,” and “shoot to red but simulate shooting to green.” Penalty takers received the instructions before each trial. They performed fifteen penalty kicks per condition. The height of the penalties was not mentioned in the instructions.

### Data Analysis

#### Penalty Outcome per Height Category

To measure the horizontal and vertical directions of the ball for a particular trial, we first determined the frame from the standard video at which the ball touched the tissue just after the goal line or was contacted by the goalkeeper (hereafter referred to as the end of the trial). The horizontal and vertical coordinates, referred to as *x* and *y* coordinates, were then measured on the screen and transformed to real-world distances. The penalties were categorized as low, medium, or high according to the *y* coordinate (low: 0 < *y* < 81.3 cm; medium: 81.4 < *y* < 162.7 cm; high: 162.8 < *y* < 244 cm). Our first set of analyses concerned the efficacy of the penalties as a function of the three height categories.

#### Kinematics of Penalty Taker

The second set of analyses determined the value of several kinematic variables computed from the markers on the penalty takers in predicting the penalty heights (the non-categorized *y* coordinates). This was done with product-moment correlations. The variables that were selected for presentation in this article were: the dominant foot height, the dominant foot speed, the dominant foot angle (the angle between a vector orthogonal to the floor and the imaginary line connecting the dominant foot markers), the non-dominant foot distance (the horizontal distance between the marker on the font part of the foot and the imaginary line that is parallel to the goal line and that crosses the penalty kick mark), the shoulder-hip-wrist angle for the dominant foot side, and the trunk angle (the angle between a vector orthogonal to the floor and the segment from the middle between the two hip markers to the middle between the two shoulder markers).

#### Timing of Saving Action

For the third set of analyses we digitized the standard videos from one frame before the moment of ball contact until the frame corresponding to the end of the trial. These analyses included the penalties (a) that were classified as low or high, (b) in which the goalkeeper dove to the side of the kicked ball, and (c) in which the goalkeeper clearly intended to stop the ball with both hands. In total, 88 penalties fulfilled those conditions. For those penalties, the *x* and *y* positions of both hands were determined and averaged over the two hands, obtaining one trajectory per trial. Those trajectories, which differed in length in terms of frames for penalties with different flight durations, were aligned with respect to the end of the trial. The reported analyses were run on the hand trajectories that were further averaged, for each kicker, per side (left, right) or per height (high, low).

## Results

### Penalty Outcome per Height Category

Table 1 presents the percentage of penalties, for each penalty taker and averaged over all penalty takers, that fell in each of the height categories. Overall, 31.3% of the penalties fell in the low category, 36.0% in the middle category, and 32.7% in the high category. Table 2 presents the percentages of penalties for each of the height categories separated for outcome. A chi-square test showed that ball height and outcome are associated variables: χ^2^(2, *N* = 638) = 24.40, *p* < 0.001. Scored penalty kicks were above the level of 33.3% for high penalties (residual = 4.1), whereas scored penalties were below that level for medium penalties (residual = −4.5).

**TABLE 1:** Percentages of penalties per height category.

	**Ball height**		
	**Low**	**Medium**	**High**
**Penalty Taker**			
1	34.0	35.8	30.2
2	23.7	43.6	32.7
3	35.1	42.1	22.8
4	26.9	38.5	34.6
5	33.4	31.2	35.4
6	38.6	38.6	22.8
7	26.8	28.6	44.6
8	9.8	37.3	52.9
9	44.1	30.5	25.4
10	27.1	35.4	37.5
11	50.0	25.0	25.0
12	25.9	44.4	29.7
Average	31.3	36.0	32.7
*n*	200	230	208

**TABLE 2:** Percentages of penalties per height category separated for goal and no goal.

	**Ball height**					
	**Low**		**Medium**		**High**	
	**Goal**	**No goal**	**Goal**	**No goal**	**Goal**	**No goal**
**Penalty taker**						
1	33.3	35.7	33.3	42.9	33.3	21.4
2	26.1	11.1	41.3	55.6	32.6	33.3
3	32.6	42.9	39.5	50.0	27.9	7.1
4	23.0	38.5	38.5	38.5	38.5	23.0
5	38.9	16.6	27.8	41.7	33.3	41.7
6	34.1	53.8	38.6	38.5	27.3	7.7
7	21.1	38.9	21.1	44.4	57.8	16.7
8	12.8	0.0	23.1	83.3	64.1	16.7
9	47.6	35.3	31.0	29.4	21.4	35.3
10	33.3	0.0	25.7	77.8	41.0	22.2
11	54.3	38.5	17.1	46.2	28.6	15.3
12	28.2	20.0	30.8	80.0	41.0	0.0
Average	32.1	27.6	30.7	52.4	37.2	20.0
*n* (%)	153 (24.0)	47 (7.4)	149 (23.3)	81 (12.7)	177 (27.7)	31 (4.9)
*n* (%) of saves	33 (16.5)		77 (33.5)		19 (9.1)	

Given these results, it becomes important to clarify the reasons for which the medium category registered more than the expected number of missed penalty kicks. For this purpose, the outcome variable was considered in three categories: 1 = save; 2 = goal despite goalkeeper touching the ball; and 3 = goal without goalkeeper touching ball. The result of a chi-square test was: χ^2^(4, *N* = 638) = 45.83, *p* < 0.001. Saves by goalkeepers were particularly frequent for the medium category (residual = 6.3). This finding was further supported by an analysis that included only the subset of the trials in which the lateral ball direction and goalkeeper’s dive direction were identical. In this case the chi-square test showed that: χ^2^(4, *N* = 364) = 54.09, *p* < 0.001, with an adjusted residual of 6.5 for saved penalty kicks at medium ball height.

Additional analyses were performed focusing on the saved penalty kicks. The numbers of saved penalty kicks for the low (*n* = 33), medium (*n* = 77), and high (*n* = 19) categories were taken as a percentage of the number of penalty kicks directed to each of these height categories (200 for low, 230 for medium, and 208 for high). As shown in the bottom row of Table 2, the percentage of saves was higher for penalty kicks with a medium height (33.5%) than for low and high penalty kicks (16.5 and 9.1%, respectively).

### Kinematics of Penalty Taker

**FIGURE 1:**
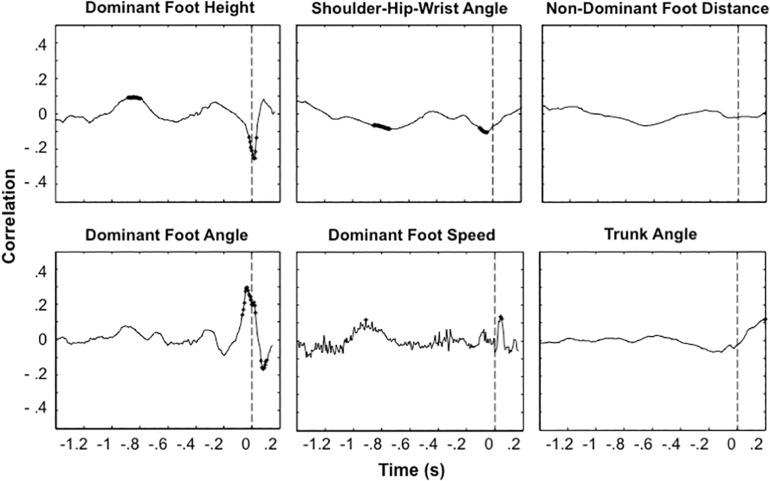
Time-evolution of correlations between single kinematic variables and ball height. Each panel gives the results for one kinematic variable. Curves represent correlations computed per penalty taker and averaged over the twelve penalty takers. The moment of ball contact is indicated with dashed vertical line segments. Asterisks indicate significance levels of *p* < 0.05 obtained with *t* tests computed on the Fisher *z* transformations of the correlations for each penalty taker, testing whether the correlations differed from zero.

The time-evolution of the correlations between the candidate kinematic variables and height are presented in Figure 1. Earlier than about 0.1 s before ball contact, the relations between the kinematic variables and height were weak or non-existent. Around the moment of ball contact, the kinematic variables that correlated with ball height were the dominant foot height and the dominant foot angle. The correlations for these variables differed significantly from zero in that period. However, with average correlations of at most about *r* = 0.3, the individual kinematic variables (as registered by us), explained less than 10% of the variance in height.

### Timing of Saving Action

**FIGURE 2:**
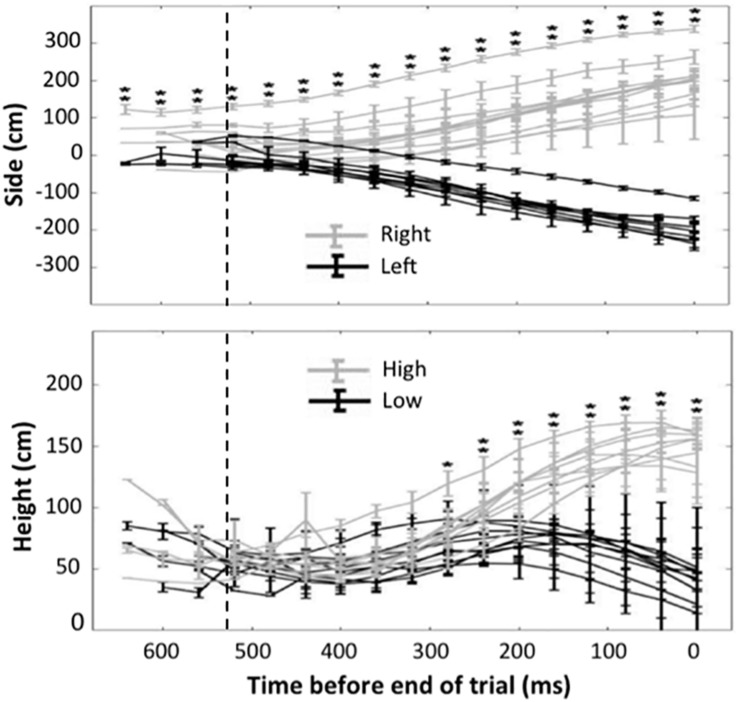
Mean horizontal position of the goalkeepers’ hands for left and right penalties (upper panel) and mean vertical position for high and low penalties (lower panel) as a function of the time until the ball reaches the goal. The results of single-tailed *t* tests on the average positions per penalty taker are indicated in each panel with asterisks. One asterisk means *p* < 0.05; two asterisks means *p* < 0.01. The dashed vertical lines indicate the average moment of ball contact.

The average ball flight time for the 88 penalties that were analyzed in this subsection was 525 ms (*SD* = 64). The mean horizontal position of the ball at the end of the trials was −227 cm (*SD* = 64) for penalties shot to the left and 233 cm (*SD* = 81) for penalties shot to the right. The upper panel of Figure 2 shows the horizontal hand position of the goalkeepers for penalties shot to the left and right as a function of the time before the end of the trial. As shown by the asterisks, the hand positions already differed significantly for left and right penalties at the first video frame that was included in the analyses (i.e. 640 ms before the end of the trial and hence 115 ms before the average moment of ball contact). The mean height of the ball at the end of the trials was 37 cm (*SD* = 24) for low penalties and 203 (*SD* = 27) for high penalties. The lower panel of the figure shows that the hand positions differed significantly for low and high penalties from 280 ms before the end of the trial until the end of the trial. With mean flight durations of 525 ms, this indicates that the height difference became evident around 245 ms after ball contact.

## Discussion

### Penalty Outcome per Height Category

A first key issue to consider is the preference of penalty takers in what concerns the height of the kicks. The percentages of penalties directed to the different height categories that we observed were: 31.3% for low penalties, 36.0% for medium penalties, and 32.7% for high penalties. Bar-Eli and Azar (2009) analyzed 311 penalty kicks from professional leagues and championships of national teams and reported 56.6, 30.4, and 12.9%, respectively, for low, medium and high penalties. Hence, we observed more high penalties and less low penalties. This difference may be related to the psychological pressure on the penalty takers, which was much lower in our study than in the one of Bar-Eli and Azar (2009). For instance, Navia et al. (2019) found that penalty kickers placed the shots higher when psychological pressure is reduced, showing that, under high pressure, penalty takers may want to avoid the risk of shooting penalties too high.

For the percentages of penalties in which goalkeepers prevented a goal, we observed 16.5% for low, 33.5% for medium, and 9.5% for high penalties. For the same categories, Bar-Eli and Azar (2009) reported 19.8, 12.6, and 0.0%, respectively. Our results hence replicate (a) that goalkeepers’ possibilities to save penalties depend on the height of the penalties and (b) that high penalties are difficult to save. Despite this broad similarity, we observed more saves overall and relatively more saves in the high and medium areas. In this regard, it may be relevant to highlight that the experiment included senior field players in close collaboration with the coach of the senior team, with junior goalkeepers of the same football club. According to all experimenters, it was easy to observe that this situation was extremely motivating especially for the goalkeepers.

### Kinematics of Penalty Taker

We next addressed the relation between the penalty takers’ movements and the height of the penalties. The observed correlations were never higher than about 0.3. The laws of physics mandate that the biomechanics of the kicker should determine the direction of the ball. This means that the moderate or low correlations that we obtained should at least partly be attributed to issues such as the selection of the analyzed variables or the precision of our measurements. The low correlations are revealing, however, if one compares them with the fact that the same measurements and the same type of analysis led to substantially higher correlations for the horizontal direction (Lopes et al., 2014). For the horizontal direction, the correlations were well above 0.8 for the dominant foot angle, hip angle, and dominant foot movement direction [see Figure 4 of Lopes et al. (2014)]. Such high correlations are in line with the high reliability of perceptual variables for the horizontal direction that has been observed in other studies (Franks and Harvey, 1997; Diaz et al., 2012). The results of the present study therefore provide evidence for the common claim that the biomechanical predictors are more subtle for height than for the horizontal direction, and are hence most probably more difficult to detect and use (Savelsbergh et al., 2002; Causer et al., 2017).

The variables that correlated moderately and significantly with height were the dominant foot height and the dominant foot angle. The negative correlations for dominant foot height indicate that the higher the foot the lower the ball direction, as stated in the literature for this variable (Prassas et al., 1990; Asai et al., 2005). For the dominant foot angle, a higher angular value (a less vertical foot position) corresponded to a higher ball trajectory. Although this is the expected direction, the significant correlation may be surprising in the sense that a similar effect did not reach significance in the study by Prassas et al. (1990) We did not observe a significant correlation for the trunk angle and hence did not obtain evidence in favor of the common recommendations about trunk angle by coaches and the opinions expressed by football players (Williams and Burwitz, 1993).

### Timing of the Saving Action

We believe that the main contribution of this article concerns the differentiated timing for the horizontal and vertical aspects of the goalkeeper saves. In this sense our research is aligned with the approach forwarded by van der Kamp et al. (2018). These authors argued that research on the penalty situation has “disproportionally focused on understanding the informational basis of spatial control (p. 170).” Although we did not address the informational basis of the temporal control, we agree with van der Kamp et al. (2018) that research on the penalty kick has had a too limited focus, and we have aimed to broaden the scope with an analysis of height in addition to side, and with an emphasis on the temporal aspects of the goalkeepers’ control in both dimensions.

With respect to the horizontal direction, the hand positions evidenced that goalkeepers took the shot direction into account more than 640 ms before the ball reached the goal, which corresponds to more than about 115 ms before the moment of ball contact. This is consistent with previous findings (e.g. Kuhn, 1988; McMorris and Hauxwell, 1997; Dicks et al., 2010). A contribution of our research is that we demonstrated the finding with a novel methodology, focusing on the actual movements of the goalkeeper rather than on verbal judgments or gaze behavior. Reviews indicate that the majority of studies on goalkeeper behavior in the penalty situation have analyzed judgments or gaze behavior of participants who observed previously recorded videos of penalty takers (Lopes et al., 2008; see Dicks et al., 2010; cf. van der Kamp et al., 2018, for one of the exceptions).

With respect to the vertical direction of the kicks, the goalkeeper positions diverged for low and high penalties around 280 ms before the end of the trial. At that moment, on average, the ball had been in flight for about 245 ms. Given a small perceptual-motor delay (55–130 ms, Lee et al., 1983; 80–144 ms, Franks and Harvey, 1997), such a timing allows goalkeepers to rely on the ball trajectory. Moreover, the low predictive value of the kinematics of the kicks with respect to height seems to make the use of trajectory information the better option. Consistent with the previous findings, Navia et al. (2017) showed that for 6 and 10 meters futsal penalty kicks the average start of hand movements toward the ball trajectory started 188 and 212 ms after ball contact respectively. These results allow the possibility of guiding the hands toward the vertical direction of the ball using ball trajectory information. The use of trajectory information is also consistent with our results concerning the penalty outcomes. This is so because more saves are to be expected at middle heights if goalkeepers initiate their actions aiming for middle heights and then later adapt the actions on the basis of height information from the ball trajectory.

### Gaze Direction and Information Usage

A final line of evidence that supports the different types of control – on the basis of the biomechanics for the horizontal direction and on the basis of the ball trajectory for height – can be found in the literature on the gaze direction of goalkeepers. In their training method, aimed at novice goalkeepers, Savelsbergh et al. (2010) proposed a standard fixation pattern from the initiation of the run-up until ball contact. Based on previous studies on the gaze behavior of goalkeepers (Savelsbergh et al., 2002, 2005), Savelsbergh et al. (2010) argued that goalkeepers should start fixating the head of the penalty taker and then lower their gaze, first to the trunk and hip region and then to the leg-foot region. Such a fixation pattern until ball contact is compatible with the claim that goalkeepers control the horizontal direction of their saving action, which is initiated before ball contact, at least partly on the basis of the biomechanics of the kicker.

Around ball contact, association football goalkeepers fixate the ball region more frequently than earlier during the run-up (Savelsbergh et al., 2002, 2010), and this is more so when they actually perform the saving action than when they merely observe the penalty taker (Dicks et al., 2010b; cf. Navia et al., 2013). Less is known about the gaze behavior during the ball flight. Although they considered a different sport, for the ball flight phase it is tempting to consider the above-mentioned study on futsal by Navia et al. (2017). These authors showed that, for 6 and 10 m penalty kicks, during the run-up futsal goalkeepers frequently fixate the body of the penalty taker. Shortly before ball contact, the variation in fixation behavior among goalkeepers is reduced, and the fixation invariantly turns to the ball. For the 10 m kick, Navia et al. (2017) claimed that “goalkeepers tended to track the ball trajectory via a smooth pursuit gaze pattern” (p. 791). Given our results concerning timing, if such results would be true also for association football goalkeepers, they would provide an additional piece of evidence for the claim that the height aspect is indeed adjusted on the basis of the detected ball trajectory.

## Perspective

The main finding of our study is that the position of the hands of the goalkeeper diverge for low and high penalties about 245 ms after ball contact, in contrast to left and right penalties, for which the divergence is more than 115 ms before ball contact. Assuming a short perceptual-motor delay (Lee et al., 1983; Franks and Harvey, 1997), this means that goalkeepers can track the first part of the ball flight for the guidance of the vertical aspects of their movements. The study also demonstrates that the vertical direction of penalty kicks is more difficult to predict from the biomechanics of the kicker than the horizontal direction. In addition, in our study the trunk orientation did not predict the height of the penalty kicks. These results are to a large extent consistent with previous findings (Savelsbergh et al., 2002; Causer et al., 2017).

A main practical implication of our research is that it is not appropriate to focus the training of goalkeepers on the detection of the trunk orientation of the penalty taker in order to predict the height of the kick (Williams and Burwitz, 1993). As an alternative, we believe that goalkeepers’ performance may be improved by concentrating on the biomechanics of the penalty taker for the horizontal direction of the kick, and on tracking the ball after contact in order to guide the vertical aspects of the movement (in line with the observations by Navia et al., 2017, for the 10-m kicks in futsal). In other words, future research should test the hypothesis by Williams and Burwitz (1993) that training programs focusing on the first part of the ball flight may improve the anticipation of the height of penalty kicks.

## Data Availability Statement

The datasets generated for this study are available on request to the corresponding author.

## Ethics Statement

The studies involving human participants were reviewed and approved by the Comité de Ética de la Investigación de la Universidad Autónoma de Madrid. The patients/participants provided their written informed consent to participate in this study.

## Author Contributions

All authors conceived the ideas expressed in this manuscript, involved in the data analyses and manuscript writing, and approved the final version of the manuscript. AH-H, DT, JI-G, and DJ digitized the regular video recordings.

## Conflict of Interest

The authors declare that the research was conducted in the absence of any commercial or financial relationships that could be construed as a potential conflict of interest.
